# Prediction of Temperature and Loading History Dependent Lumbar Spine Biomechanics Under Cyclic Loading Using Recurrent Neural Networks

**DOI:** 10.1007/s10439-022-03128-3

**Published:** 2023-01-28

**Authors:** Nadja Blomeyer, Saurabh Balkrishna Tandale, Luis Fernando Nicolini, Philipp Kobbe, Thomas Pufe, Bernd Markert, Marcus Stoffel

**Affiliations:** 1 Institute of General Mechanics, RWTH Aachen University, Eilfschornsteinstr. 18, 52062 Aachen, Germany; 2 Department of Trauma and Reconstructive Surgery, University Hospital RWTH Aachen, Pauwelsstraße 30, 52074 Aachen, Germany; 3 Department of Anatomy and Cell Biology, University Hospital RWTH Aachen, Pauwelsstraße 30, 52074 Aachen, Germany

**Keywords:** Viscoelasticity, Spine, Biomechanics, Recurrent Neural Network, Loading-history

## Abstract

Extended-duration cyclic loading of the spine is known to be correlated to lower back pain (LBP). Therefore, it is important to understand how the loading history affects the entire structural behavior of the spine, including the viscoelastic effects. Six human spinal segments (L4L5) were loaded with pure moments up to 7.5 Nm cyclically for half an hour, kept unloaded for 15 min, and loaded with three cycles. This procedure was performed in flexion-extension (FE), axial rotation (AR), and lateral bending (LB) and repeated six times per direction for a total of 18 h of testing per segment. A Long Short-Term Memory (LSTM) Recurrent Neural Network (RNN) was trained to predict the change in the biomechanical response under cyclic loading. A strong positive correlation between the total testing time and the ratio of the third cycle to the last cycle of the loading sequence was found (BT: $\tau$ =  0.3469, *p* = 0.0003, RT: $\tau$ =0.1988, *p*  =   0.0377). The moment-range of motion (RoM) curves could be very well predicted with an RNN ($R^2$=0.988), including the correlation between testing time and testing temperature as inputs. This study shows successfully the feasibility of using RNNs to predict changing moment-RoM curves under cyclic moment loading.

**Supplementary Information:**

The online version contains supplementary material available at 10.1007/s10439-022-03128-3.

## Introduction

Lower back pain (LBP) is one of the pathological sources most frequently treated in our society. About two-thirds of all adults suffer from LBP during their life.^24^ Studies have shown a correlation between repetitive motions and forces with increased risk factors for work-related musculoskeletal disorders (WMSD), especially in the lower back.^5^ The loading conditions acting on the spine, therefore, play an important role in the development of these disorders.^6^ The spine and especially the intervertebral disc (IVD) exhibit a changing structure-mechanical behavior during the day, based on an interaction of multiple factors like osmosis, relaxation, creep, and fluid loss.^15^ To optimize the practical work routine and predict its influence on WMSD it is important to understand the material behavior of the spine under cyclic loading. Therefore, other studies have dealt with the dependency of different factors on the lumbar spine material behavior. Multiple studies determined the influence of the degeneration degree of cadaveric specimens on their Range of motion (RoM) curve characteristics.^1,9^ Shojaei *et al*.^24^ investigated the change in the viscoelastic response of the lower back on passive momentary loading *in vivo* with the age of the participants. A change of the stiffness of the spine *in vitro* was noted with the testing temperature^2,25^ and an increased exposure time dependent on the moisturizing conditions.^30^ Spinal segments are discovered to become stiffer with an increased loading rate.^9^ Multiple studies analyze the viscoelastic properties under cyclic loading and determined parameters for material models from *in vitro* and *in vivo* experimental results.^8,18,27^ Stolworthy *et al*.^25^ found a formulaic connection predicting the RoM curve with changing loading rates. The use of Artificial Neural Networks (ANNs) for classification tasks like the detection of pathologies or specific patterns in different data sources is a rising field of interest in mechanics^26^ and especially biomechanics.^7,23^ Fewer studies use ANNs trained with experimental data to predict difficult accessible parameters, e.g. facet joint loads^22^ or the total displacement of bones under cyclic loading.^19^ The predictions of these studies show promising results, as they can access parameters, which are dependent on numerous variables. Alternative, more established methods like Finite Element Analysis (FEA) are limited by the need for a material model. Here, assumptions regarding the material properties have to be made in order to provide *in silico* results.^20^ Whereas, the data-driven nature of ANNs can be used to predict the material response independent of the complexity of the material itself. This was shown in a study by Rahmanpanah *et al*.^21^ successfully predicting load-displacement curves of long bone under cyclic loading using ANNs and emphasizing the suitability for modeling nonlinear material behavior. The application of ANNs in order to predict the change in moment-RoM curves of spinal segments under cyclic loading, however, has not been performed before. This utilization is specifically interesting, as this behavior of spinal segments depends on multiple factors. Particularly the variability between specimens and high computational costs of complex material models are problems, which could possibly be solved using ANNs. Therefore, the aim of this study can be divided into two parts. First, the change in structural biomechanical behavior under cyclic moment loading of human spinal segments will be assessed including the effect of temperature and exposure time. Second, these results will be used to predict the change in RoM under cyclic loading using ANN.

## Methods

### Experimental Investigations

#### Specimen Preparation

Six intact human lumbar spine segments (L4L5) of five female and one male donor with a mean age of 76.2 years (std 7.1) and no signs of severe degeneration were selected. The fresh-frozen lumbar spines were obtained from the Institute of Molecular and Cellular Anatomy, University Hospital RWTH Aachen (Aachen, Germany) and cut at the L5S1 and L3L4 disc. After harvesting,. the specimens were carefully dissected, removing the surrounding tissue and leaving the ligamentous structure intact. Until testing the specimen were stored in double-sealed bags at $-18\,^{\circ }$C. The specimens were divided into two groups of three specimens to be tested at room temperature (RT) and body temperature (BT). The age, gender, and test rig condition of each specimen are stated in Table 1. The vertebrae were embedded in a polymethyl methacrylate (PMMA) resin (Technovit 4004, Heraeus Kulzer GmbH, Germany). In addition, polymeric screws were screwed into the embedded vertebrae before embedding to secure a firm seating of the embedding. Dehydration was prevented by periodic spraying of the specimen with a saline solution. Before testing, the specimens were thawed overnight at $4\,^{\circ }$C.

**Table 1:** Gender of donor, donor age and testing condition for each specimen.

Specimen	Gender	Age	BT/RT
1	f	83	BT
2	f	80	BT
3	f	64	BT
4	m	81	RT
5	f	80	RT
6	f	69	RT

#### Test Set Up

The specimens were tested under pure moment loading in all three directions under physiological environment using previously reported methods.^3,4,31^ The use of an electromagnetic tracking sensor (Aurora V2, Northern Digital Inc., Canada) connected firmly to the top of the embedding enabled the measurement of the motion of the upper vertebrae without visual sight. The specimens were tested in a humidified chamber, which was kept at $37\,^{\circ }$C for the specimens tested at BT.

#### Testing Protocol

**Figure 1:**
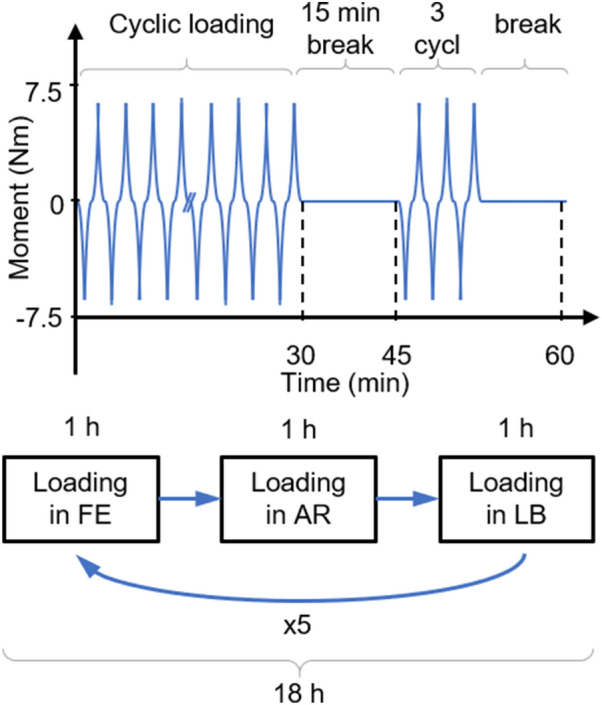
Schematic description of the loading protocol performed on each specimen. *FE* Flexion-Extension, *LB* Lateral Bending, *AR* Axial Rotation.

All specimens were preconditioned with an axial load of 500 N for 15 min to avoid additional, freezing-induced hydration. To assure the adaption of the specimen to the environmental temperature inside the bioreactor, a waiting time of 25–30 min after closing the bioreactor was applied.^11^ Subsequently, each specimen was tested for 18 h following the procedure described in Fig. 1. To model the diurnal loading-unloading ratio of 2:1, a sequence of 30 min of cyclic loading was performed before 15 min of holding the specimen in the neutral position (break).^28^ Subsequently, three loading cycles were performed, to determine the RoM after the break. One hour after the start of this procedure the sequence was started for another loading direction in the order of Flexion-Extension (FE), Axial Rotation (AR), and Lateral Bending (LB) and performed for every loading direction six times. The loading cycles were continuously applied with an angular velocity of 1 $^{\circ }$/s up to a moment of 7.5 Nm in the direction of loading.

#### Analysis

MATLAB (version R2019b, The MathWorks, Natick, MA) was used to perform the analysis and the statistical evaluation of the experimental data. The RoM curve of each loading cycle was evaluated according to a previously established procedure.^3^ The sensor was used as a reference to track the loading direction. The RoM was defined as the angular displacement of the sensor regarding its starting position projected into the plane perpendicular to the loading direction. The first cycle of every loading sequence was considered as preconditioning and therefore excluded from further analysis. According to the Shapiro–Wilk test, normal distribution of the data was not given. Hence, the RoM medians of the specimen for each loading sequence at BT and RT were calculated. The relation $r_{\text {break}}$ between the absolute RoM at the last cycle before the break, $RoM_{30{\text {min}}}$, and the third cycle after the break, $RoM_{3,{\text {break}}}$ (Eq. (1)), as well as the relation $r_{\text {load}}$, between the absolute RoM at the third cycle, ${RoM_3,{\text {start}}}$, and $RoM_{30{\text {min}}}$ (Eq. (2)) were investigated.

1 $$\begin{aligned} r_{\text {break}} \,=\, \frac{RoM_{30{\text {min}}}}{RoM_{3,{\text {break}}}} \end{aligned}$$

2 $$\begin{aligned} r_{\text {load}} \,=\, \frac{RoM_{3,{\text {start}}}}{RoM_{30{\text {min}}}} \end{aligned}$$

A Kendall’s $\tau$ Correlation Analysis was performed for the total testing time and $r_{\text {load}}$. Fisher’s transformation was used to calculate the difference between the correlation factors for RT and BT. A level of statistical significance of $\alpha$  =  0.05 was assumed. To investigate the differences in the progression of the curves between the different testing conditions, the Wilcoxon Signed Rank Test, to test the difference between the RoM of the spine at RT and BT, the Mann–Whitney U-Test was used.

#### Preparation of Input Data

All moment-RoM curves were parameterized with two modified Boltzmann sigmoid functions introduced by Zirbel *et al*.^32^ with Eq. (3).

3 $$\begin{aligned} RoM \,=\, \frac{A}{1+e^{a_1(m-m_1)}}-\frac{B}{1+e^{a_2(m-m_2)}}+B \end{aligned}$$

**Figure 2:**
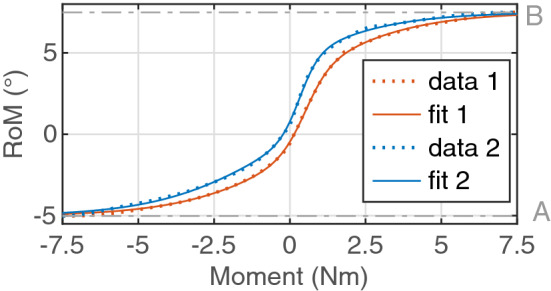
Exemplary visualization of the fitting procedure. The parameters *A* and *B* are defined as the minimum and maximum values of the *RoM*.

Hereby, *A* was defined as the minimum and *B* as the maximum *RoM* value of each curve, $a_1$, $a_2$, $m_1$, and $m_2$ were optimized with the Nonlinear Least Squares approach. A fit of the curve with an $R^2$ value higher than 0.985 was considered as sufficient. An exemplary visualization of the fitting procedure is shown in Fig 2. Hence, the total moment-RoM curve was described by ten parameters. In order to multiply the existing data, it was taken advantage of the symmetric mechanical behavior in the sagittal plane of the spine. The RoM curves in LB and AR were additionally flipped. In that way, 179 sequences with the parametric RoM curve data of each cycle in the first 30 min of cyclic loading were created.

#### Surrogate Model for Moment-RoM Curve Prediction

**Figure 3:**
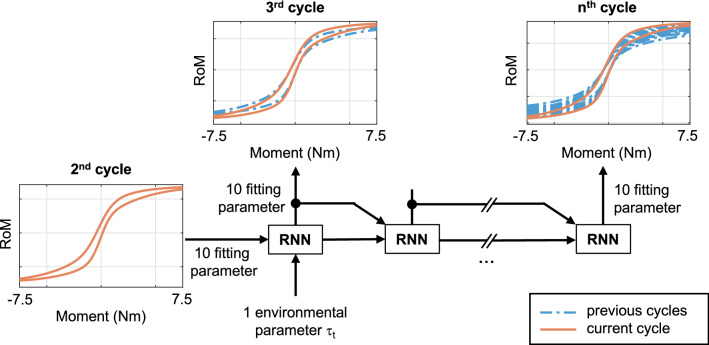
Overall computational graph for the recurrent neural network (RNN) used in this study. 10 fitting parameters describing the experimental moment-RoM curve of the spine during the 2nd cycle and the parameter $\tau _{\text {t}}$ defining the environmental conditions are used as input parameters. The RNN predicts 10 fitting parameters representing the moment-RoM curve for the following cycles. The change in the curve over the different cycles is shown schematically with a red line describing the current predicted time step and a blue dotted line for the previous time steps.

As inputs, the fitted curve parameters representing the experimental moment-RoM curve of the second cycle and Kendall’s $\tau$ Coefficient multiplicated with the total testing time, $\tau _t$, were used. The ten fitting parameters describing the curves of the following cycles were defined as the outputs of the ANN (Fig 3). In this way, the hysteresis curve of each cycle can be generated using Eq. (3).

The current research uses the following notation. The *p*th sequence is denoted by a superscript [*p*], the *j*th step in a sequence is denoted by superscript $\{j\}$, an input sequence is represented as

4 $$\begin{aligned} x^{[p]} \,=\, [(\tau _t, A, B, a_1^1, m_1^1..,m_2^2)^{\{1\}},\ldots ,(\tau _t, A, B, a_1^1, m_1^1..,m_2^2)^{\{j\}},\ldots , \\ (\tau _t, A, B, a_1^1, m_1^1..,m_2^2)^{\{P\}}] \end{aligned}$$

where, the superscript 1 refers to the upper curve and 2 to the lower curve of the hysteresis and $\{P\}$ denotes the last time-step of the mentioned sequence, and finally the *p*th output sequence is represented as $y^{[p]}$. An ordered pair of the *p*th input and output sequence can be written as follows $(x, y)^{[p]}$.

To, give the ANN numerical stability and circumvent convergence issues we adopt feature scaling to the collected input and output sequences. For each input and output feature the following linear transformation is applied.

5 $$\begin{aligned} {\chi }^{n} \,=\, \frac{\chi - \chi _{\text {m}}}{\chi _{\text {d}}} \end{aligned}$$

where,

6 $$\begin{aligned} \chi _{\text {m}} \,=\, \frac{\chi _{\text {max}} + \chi _{\text {min}}}{2} ~~~~\text {and}~~~~ \chi _{\text {d}} \,=\, \frac{\chi _{\text {max}} - \chi _{\text {min}}}{2} \end{aligned}$$

In Eq. (6), $\chi _{\text {max}}$ and $\chi _{\text {min}}$ represent the maximum and minimum values of the feature under consideration and the transformation in Eq. (5) maps the features to the range $[-1, 1]$. Thus, the scaled ordered pair of input and output sequences are represented as $[{\varvec{x}}^n, {\varvec{y}}^n]^{(p)}.$ The proposed surrogate model belongs to the class of ANNs and can be mathematically described as

7 $$\begin{aligned} {\mathcal {F}}_{NN} : {\varvec{x}}^n \Longrightarrow \tilde{{\varvec{y}}}^{n} \end{aligned}$$

Since the parameters of the ANNs are initialized randomly at first, it requires tuning to obtain the desired approximation of the output. This process of tuning is referred to as training and is discussed in Section 2.3 below. Also, for simplicity, we drop the superscript *n* corresponding to the scaled variables and agree that only normalized quantities are provided for training and prediction through the ANN.

### Preliminaries on Neural Networks

This section gives a brief introduction to the underlying concept of ANNs describing the fully connected neural network and build towards the formulation of a Gated Recurrent Neural Network (RNN).

#### Fully Connected Neural Networks

ANNs consist of interconnected units termed neurons and are usually arranged in the form of sequential stack of layers. Each neuron from every layer receives input in the form of a weighted sum from all neurons in the previous layer. The information can also be processed in the form of product units from the previous layer. However, with the present research, we utilize the weighted sum procedure. Further, the processed information is passed through an activation function, which controls the strength of the output from each neuron. A mathematical representation of the fully connected forward transformation is given as follows.

8 $$\begin{aligned} {\varvec{z}} \,=\, {\varvec{W}}~{\varvec{s}} + {\varvec{b}}~~~ \text {followed by,}~~ {\varvec{t}} \,=\, \varphi ({\varvec{z}}) \end{aligned}$$

**Figure 4:**
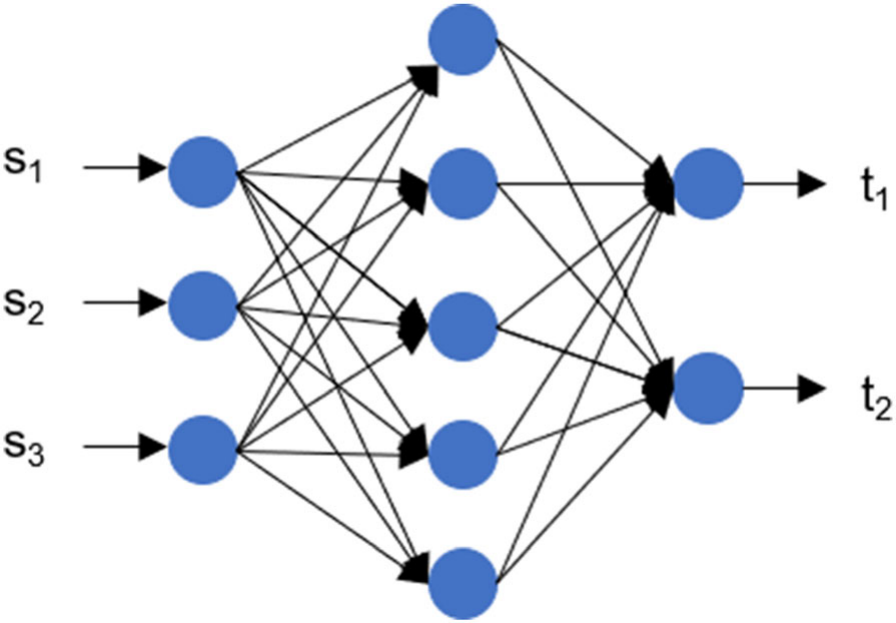
A single hidden layer fully connected neural network. The network accepts a vector ${\varvec{s}}$ as the input and approximates a relationship to compute the output vector ${\varvec{t}}$ using training algorithm.

In the above equation, the term ${\varvec{s}}$ represents the input to the fully connected layer, ${\varvec{W}}$ and ${\varvec{b}}$ represent the corresponding weights and the biases of the connection, $\varphi$ represents a scalar activation function that is applied element-wise on the weighted output ${\varvec{z}}$, and ${\varvec{t}}$ represents the activated output. This concludes the fundamental transformation available in ANNs termed the feed-forward transformation or the fully connected transformation. The parameters of the transformation are optimized using a supervised backpropagation algorithm e.g., the gradient descent where an employed error function such as the mean squared error (MSE) is minimized to obtain appropriate parameter tuning across all features. A simple one-layer network corresponding to the transformation mentioned in Eq. (8) can be seen in Fig. 4. The convergence of the training depends highly on the hyperparameters employed for the ANN. These hyperparameters correspond to the number of hidden layers, the number of hidden neurons, the learning rate employed, the number of batches handled in parallel to update the parameters, and the task under consideration. These hyperparameters are tuned manually (See “Hyperparameter Tuning” section), while the tuning of the parameters (Weights and Biases) is handled automatically by the employed learning algorithm. Once the neural network is trained until the desired MSE value is reached, they act as good function approximators,^13^ however, the number of parameters increases quadratically with the number of hidden layers. This affects the computational speed while training and hampers the ability of the network to have long-term dependencies. With the advent of deep learning,^17^ more advanced transformations like convolution and recursion circumvent the aforementioned disadvantages by parameter sharing^29^ and adapting topologies to account for long-term dependencies.^12^

#### Gated RNN: Long-Short Term Memory (LSTM)

In the previous subsection, the fundamental fully connected transformation was introduced by establishing the forward pass of an ANN. Further, to account for long-term path dependencies we introduce an RNN known as the Long Short Term Memory (LSTM).^10,12^ The core concept of LSTM is the cell state and the various gates available to learn long and short-term dependencies. The gates process the input information ${\varvec{s}}^{\{i\}}$ along with the set of internal variables ${\varvec{S}}^{\{i\}} \,=\, [{\varvec{h}}^{\{i-1\}}, {\varvec{c}}^{\{i-1\}}]$ by using fully connected layers having the following parameters (${\varvec{W}}^i, {\varvec{V}}^{i},{\varvec{B}}^i$), (${\varvec{W}}^f, {\varvec{V}}^{f},{\varvec{B}}^f$) and (${\varvec{W}}^o, {\varvec{V}}^{o},{\varvec{B}}^o$). The parameters used for computing the candidate $\varvec{\widehat{c}}^{\{i\}}$ for cell update are represented by (${\varvec{W}}^c, {\varvec{V}}^{c},{\varvec{B}}^c$), where ${\varvec{W}}^{\alpha }~\epsilon ~\varvec{{\mathbb {R}}}^{n \times l},{\varvec{V}}^{\alpha }~\epsilon ~\varvec{{\mathbb {R}}}^{n \times n},~{\varvec{B}}^{\alpha }~\epsilon ~\varvec{{\mathbb {R}}}^{n}$,$\alpha \,=\, [i, o, f, c]$, *n* indicates the number units of the cell and *l* represents the length of the input and outputs sequence. The variable ${\varvec{h}}^{\{i-1\}}$ traces the working memory while ${\varvec{c}}^{\{i-1\}}$ is responsible for tracking the long-term memory of the sequential data. In the following, we present the equations to complete the forward pass of the LSTM cell.

9 $$\begin{aligned} {\varvec{g}}^{i}&\,=\, \sigma ({\varvec{W}}^i~{\varvec{s}}^{\{i\}} + {\varvec{V}}^i~{\varvec{h}}^{\{i-1\}} + {\varvec{B}}^i) \end{aligned}$$

10 $$\begin{aligned} {\varvec{g}}^{f}&\,=\, \sigma ({\varvec{W}}^f~{\varvec{s}}^{\{i\}} + {\varvec{V}}^f~{\varvec{h}}^{\{i-1\}} + {\varvec{B}}^f) \end{aligned}$$

11 $$\begin{aligned} {\varvec{g}}^{o}&\,=\, \sigma ({\varvec{W}}^o~{\varvec{s}}^{\{i\}} + {\varvec{V}}^o~{\varvec{h}}^{\{i-1\}} + {\varvec{B}}^o) \end{aligned}$$

12 $$\begin{aligned} \varvec{\bar{c}}^{\{i\}}&\,=\, \tanh ({\varvec{W}}^c~{\varvec{s}}^{\{i\}} + {\varvec{V}}^c~{\varvec{h}}^{\{i-1\}} + {\varvec{B}}^c) \end{aligned}$$

In Eqs. (9), (10), (11) the input gate ${\varvec{g}}^{i}$, the forget gate ${\varvec{g}}^{f}$, and the output gate ${\varvec{g}}^{o}$ operations are presented. It can be observed that all the gates are activated with the sigmoid function denoted by $\sigma$. This bounds the output of the gates between zero and one and is responsible for governing the data flow in the LSTM cell. Also, the candidate for cell update is computed in Eq. (12). Further, we introduce the transformation used to update the cell state. This concludes the forward pass through an LSTM cell.

13 $$\begin{aligned} {\varvec{c}}^{\{i\}}&\,=\, {\varvec{g}}^{f}\odot {\varvec{c}}^{\{i-1\}} + {\varvec{g}}^{i}\odot \varvec{\bar{c}}^{\{i-1\}} \end{aligned}$$

then for the hidden state it follows

14 $$\begin{aligned} {\varvec{h}}^{\{i\}}&\,=\, {\varvec{g}}^{o}\odot \tanh ({{\varvec{c}}^{\{i\}}}) \end{aligned}$$

finally the output vector is computed by

15 $$\begin{aligned} {\varvec{t}}^{\{i\}}&\,=\, {\varvec{g}}^{o}\odot \tanh ({{\varvec{c}}^{\{i\}}}) \end{aligned}$$

The operator $\odot$ corresponds to element-wise multiplication between the vectors seen in Eqs. (13), (14), (15) and allows the gate tensors ${\varvec{g}}^{i}, {\varvec{g}}^{f}~\text {and}~{\varvec{g}}^{o}$ to govern the data flowing to cell state ${\varvec{c}}^{\{i\}}$, forget some unwanted data, and finally pass the selected information as the output ${\varvec{t}}^{\{i\}}$.

**Figure 5:**
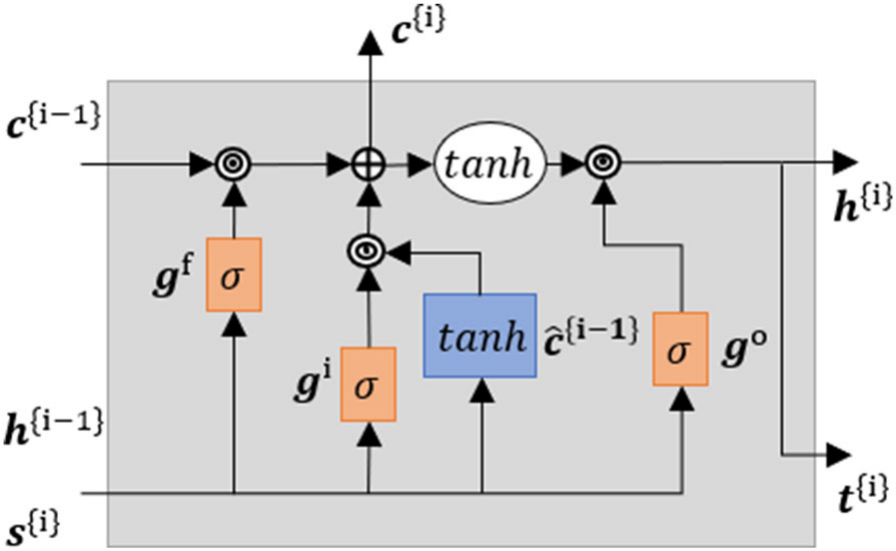
Schematical vizualization of an LSTM cell: The fully connected transformations are represented by orange and light blue rectangular blocks having sigmoid ($\sigma$) and hyperbolic tangent (tan*h*) activation.

### Training Procedure and Implementation Details

In the present study, we have a total of 179 sequences. We split the data into 116 training, 64 test, and 9 validation sequences. This split was chosen after performing a trial and error approach and observing the best mean squared error value (MSE). Each of these sequences has variable timesteps, and thus in the present study, we have implemented so-called sequence masking. During training, the input and output sequences are processed in batches, thus the samples must be padded to the same sequence length before they are used as a batch for training. These padded elements are zero and are artificial in nature. To avoid any adverse impact on the training process, a mask is employed and passed to the neural network model which ignores the padded part of the sequence and hence does not affect the training process. Furthermore, in the case of predictions, since the outputs are computed from one time step to another, such a sequence mask is not required. The network’s parameters were initialized randomly. The research aims to optimize these free parameters (weights and biases) in a way that the ANN learns the underlying function of the data. To do so, a loss function was employed which measures the error $\tilde{{\varvec{E}}}$ between the predicted output sequence $\hat{{\varvec{t}}}^{(m)}$ and the sampled output sequence denoted by ${\varvec{t}}^{(m)}$ in a mathematical form.

16 $$\begin{aligned} \tilde{{\varvec{E}}} \,=\, \frac{1}{N}\sum _{m \,=\, 1}^{N}{\varvec{t}}^{(m)}~~ \end{aligned}$$

where,

17 $$\begin{aligned} {\varvec{t}}^{(m)} \,=\, \frac{1}{l^{(m)}}\sum _{i \,=\, 1}^{l^{(m)}}{\varvec{e}}^{\{i\}}~~\text {and}~~ {\varvec{e}}^{\{i\}} \,=\, \parallel {{\varvec{t}} - \varvec{\hat{t}}}\parallel ^{2} \end{aligned}$$

Equation (17) is referred to as the mean squared error, where *N* denotes the number of sequences used for training and $l^{(m)}$ denotes the length of the *m*th sequence. The trainable parameters of the computational graphs presented in Table 2 are optimized on the aforementioned loss function by a gradient descent optimizer. The optimizer employed for the current research corresponds to the Adam optimizer.^16^ The mentioned training process of the model was performed in Python with Keras library. All computations were performed on a work station having 64 GB RAM, Nvidia GeForce RTX 3090 GPU and AMD $\text {Ryzen}^{\text {TM}}~7~5800~\text {X}$ CPU with 16 cores. Both the neural network training and prediction were performed on the GPU (Fig. 5).

**Figure 6:**
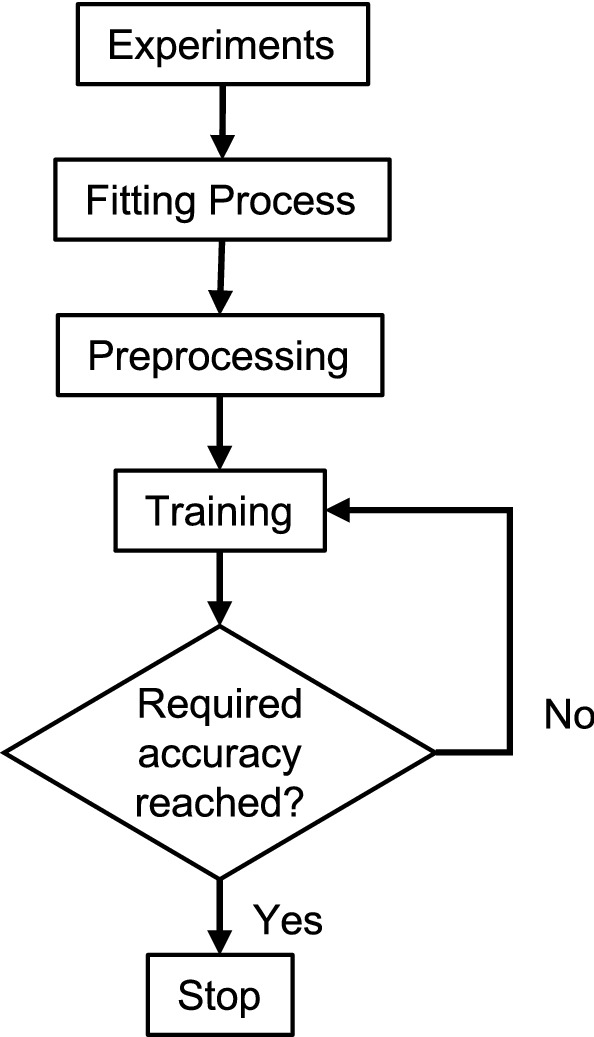
Workflow for the development of the surrogate model. Required accuracy was evaluated based on the loss value and the performance of the model on the validation data.

### Hyperparameter Tuning

The parameters that are not optimized through backpropagation algorithm are referred to as hyperparameters. These parameters are influential on the ability of the ANN to approximate an underlying function and the computational expense required for training. With the present research, we have tuned the number of units (*n*) of the LSTM cell, the number of LSTM layers utilized, and the probability of dropout. For the rest, we have employed typical hyperparameter values, as an example, the parameters of Adam optimizer take inference mentioned in Kingma *et al*.^16^ In the present research, we employ a trial-and-error strategy to tune the aforementioned hyperparameters. The tuning was performed manually by adopting an early stopping criteria and by observing the solution every 25th epoch. Furthermore, the choice for the number of epochs (*e*) required for successfully training the network was itself an additional hyperparameter and for the present work, the training was performed for $e \,=\, 44{,}753$ epochs. The early stopping method employed for this research consists of two conditions:

1. Performance of the model on the validation data.
2. The stoppage of training due to the accomplishment of desired loss value. (MSE  =  $5\times 10^{-4}$)

The first early stopping strategy is responsible for monitoring the moving averages of training and the validation error and interrupts training of the model if the solution is unlikely to converge further over 50 epochs. Once the training stops, the hyperparameters are changed and the training was resumed until both the strategies of early stopping are satisfied. This increased the efficiency of the search strategy employed for the research problem with a credible choice of the hyperparameters for further investigations. The hyperparameters optimized for the current research problem are mentioned in Table 2 (Fig. 6).

**Table 2:** Tuned hyperparameters.

Hyperparameter	Value
Number of epochs	4.4753*e*4
Batch size	324
Dropout	0.15
Learning rate	0.001
Number of hidden recurrent layers	6
Number of hidden dense layer	1
Number of hidden units	[64, 64, 128, 128, 256, 256]

## Results

**Figure 7:**
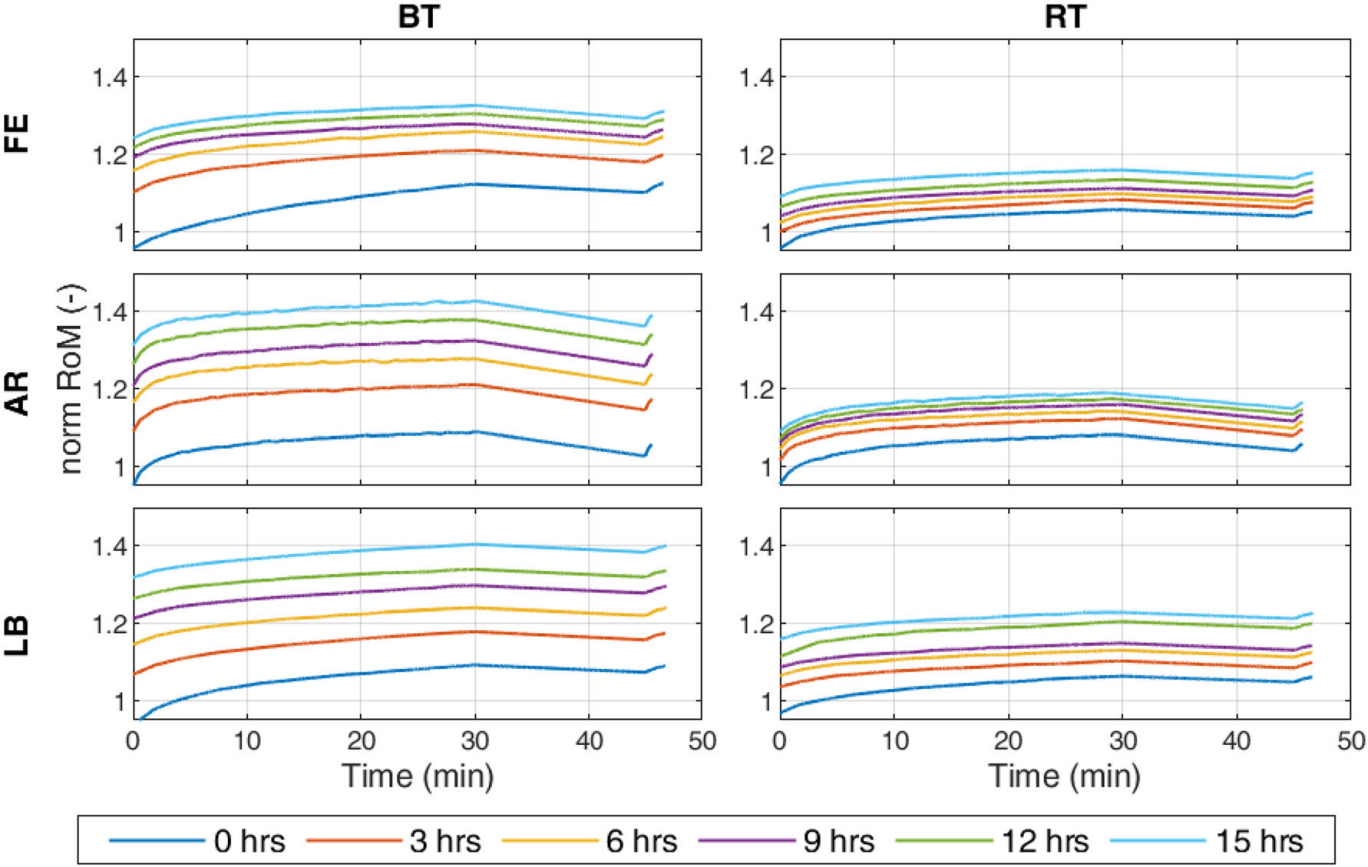
Development of the median absolute RoM of each testing sequence normalized on the third cycle of the first testing sequence in each direction comparing room temperature (RT) and body temperature (BT). The testing sequences for axial rotation (AR) and lateral bending (LB) were started 1 and 2 h after the start of flexion-extension (FE), respectively.

### Experimental Results

The median of the absolute RoM was calculated for each testing sequence, temperature, and loading direction. In 30 min of testing, approximately 20 cycles for LB and FE as well as 60 cycles for AR could be performed depending on the stiffness of each specimen. The progress of the median RoM normalized on the third cycle of the associated direction is shown in Fig. 7, in which a creep effect behavior is visible. A higher increase in RoM over the total testing time is noted for BT in comparison to RT for each direction (*p* = 0.0244). No significant differences between the different directions were noted (*p* > 0.05). After the break, the RoM did not recover to its original amount. A decreasing tendency of the creep strength was noted with the sequences with a significantly higher $r_{\text {load}}$ for the sixth sequence with a median of 0.9471 in comparison to the first with a median of 0.9176 (*p* = 0.0065). For $r_{\text {break}}$, no similar tendency was noted (*p* > 0.05, sequ. 1: 1.03, sequ. 6: 1.02). A lower $r_{\text {load}}$ and a higher $r_{\text {break}}$ for BT than for RT was proven ($r_{\text {load}}$: $p< 0.0001$, BT: 0.9351, RT: 0.9492; $r_{\text {break}}$: *p*   =   0.0263, BT: 1.0275, RT: 1.0209). A positive correlation of $r_{\text {load}}$ and the total testing time was found for BT (*p*   =   0.00028, $\tau$  =  0.3469) and RT (*p*   =   0.0377, $\tau$  =  0.1988). However, a significant difference between these correlation factors was not found (*p* > 0.05).

### Fitting Procedure

The fitting process of the moment-RoM curves shows good results for the curves in all directions (Fig. 2). $R^2$ values of less than 0.985 were preliminarily found for the first cycle of a sequence and the first cycle after the break. The fitting parameters of the 179 sequences can be found in the supplemental material. A tendency of decreasing A and increasing B values with the testing time is noted. No specific tendency was identified for the other fitting parameter.

### LSTM Modeling

**Figure 8:**
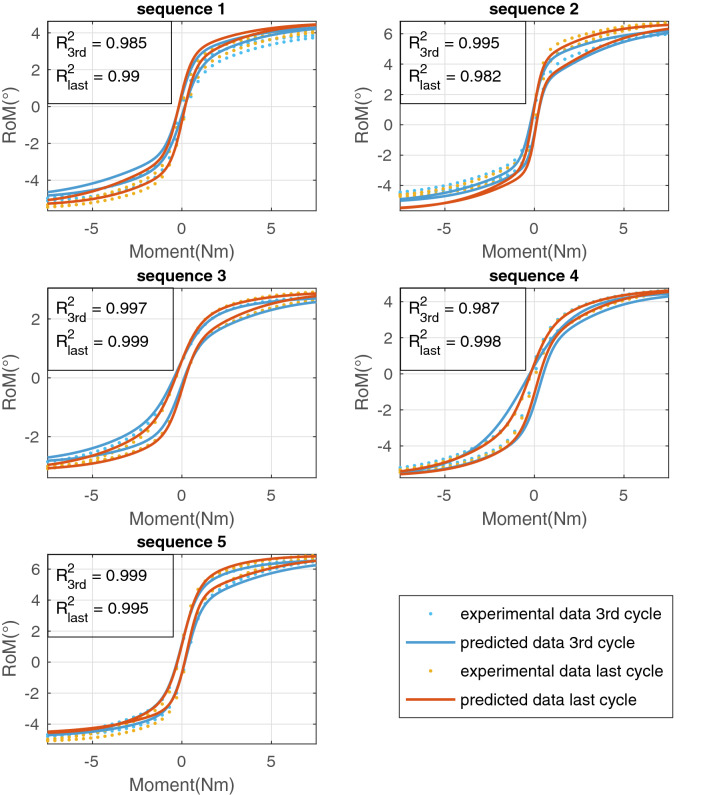
Comparison of the experimental data with the predicted moment-RoM curves of 5 sequences of the validation data set. For each sequence, the third and last tested cycles are shown and the $R^2$ values stated.

While training the RNN with $4.4753\times 10^{4}$ Epochs, we were able to achieve a mean squared error loss of $4.998\times 10^{-4}$. The predicted RoM curves of the validation data had a mean $R^2$ value of 0.988 with the experimental values (Fig. 8).

## Discussion

To the best of the authors’ knowledge, this study is the first study to predict the change in viscoelastic behavior of the human lumbar spine under pure moment cyclic loading using an ANN.

### Experimental Results

Other studies have investigated the creep behavior under cyclic loading *in vitro* and *in vivo*. In accordance with this study, an increasing laxity with the loading time was found by Gale *et al*.,^8^ when applying 20,000 cycles of low-angle flexion on degenerated human and cervine lumbar spines. However, the difference in applied cycles and degree of deformation makes it difficult to compare the amount of stiffness increase to our study. An *in vivo* study on the creep deformation of the human trunk found a creep angle increase of  13% during 12 min of repetitive flexion.^27^ We found a median increase in RoM of 8.74% in FE for the first sequence. These values stand in good agreement with each other, as the additional increase *in vivo* can be explained by the increasing laxity of the muscular structure and the additional axial loading by the bodyweight acting on the lumbar spine. Little *et al*.^18^ investigated the increase in laxity during static and repetitive flexion *in vitro* and found a significantly stronger increase for static momentary loading than for repetitive. Here, the moment is applied by pulling the cranial vertebra in the anterior direction so that the applied moment on the spine is linearly increasing in the caudal direction. Compared to our study, a low increase in RoM of 2.42% after ten cycles leads to the conclusion that the change in biomechanical behavior is strongly dependent on the loading amplitude. Due to the fact that the duration of loading was constrained by the time, the number of cycles performed per sequence was strongly dependent on the stiffness of the specimen in each direction. A lower RoM was noted for AR in comparison to LB and FE. No significant difference or tendency was found for $r_{\text {load}}$ in the different direction. This behavior could indicate a stronger dependency of the RoM change under cyclic loading on the loading time and moment loading than on the number of cycles and frequency of loading under these testing conditions. Nevertheless, the difference in loading frequency and loading cycles of the different loading directions cannot be neglected and has to be kept in mind, when evaluating the results of this study.

After 20 min of relaxation in the neutral position after - 40 min of loading, some recovery was noted in the study by Little *et al*.,^18^ although the initial state was not reached (115% higher deformity). In our study, the same tendency was noted. After 30 min of loading and 15 min of unloading the RoM decreased from 107 to 104% for BT and 105 to 103% for RT. This stands in line with other studies stating that the recovery time *in vitro* is longer than *in vivo*, where the relation of unloading and loading is also 1/3 to 2/3 with 8 h of unloading accounting for 16 h of loading.^14,28^ Hereby the degradation of the material *in vitro* in dependency on the environmental conditions and the exposure time is to be considered.^30^ It is a challenge to distinguish between the changes in material behavior due to creep effects and the degradation of the material. Wilke *et al*.^30^ found a with exposure time linearly increasing RoM at RT with a slope between 0.41 and 0.69 %/h. Signs of stronger decay of the biological material in sight and smell in our study lead to the conclusion, that these values increase when tested at BT. However, a higher $r_{\text {break}}$ at BT, indicates that the viscoelastic properties are stronger depending on the temperature than the degradation. This verifies better modeling of the *in vivo* material behavior at BT compared to RT. This conclusion stands in line with other studies.^2,25^ While the effect of recovery did not significantly change over the total testing time, a decreasing tendency of the creep effect was noted. This is presumed to be a result of the incomplete recovery after each testing sequence. The positive correlation between $r_{\text {load}}$ and the total testing time demonstrates an increasing $r_{\text {load}}$ and therefore a decreasing creep effect with time. It is linearly converging to unity. Although no significant difference between BT and RT was found, the prediction quality of the network was improved by the inclusion of this factor as an input. Although the biomechanical behavior *in vivo* cannot completely be modeled by *in vitro* studies, they represent the best opportunity to quantify otherwise not accessible values. Therefore, it is important to perform *in vitro* tests in physiological environments. The small sample size and the advanced donor age are limitations of this study and could have affected the results. In order to investigate less-significant hypotheses, more specimens have to be tested. The limited number of specimens also influences the training data. Even though it was possible to expand the data by flipping the curve of AR and LB using the symmetry of the spine, it was only possible to use 179 sequences from six different specimens for training. The data is limited to the L4L5 segment.

### LSTM Modeling

Once the training data was extracted, it was preprocessed and used for training the free parameters of the RNN through backpropagation. The internal parameters developed during the training process are responsible for implicitly learning the path-dependent viscoelastic behavior, thus, representing a reduced-order nonlinear counterpart of the internal parameters used in the classical continuum model. Therefore, the predictions of the RNN show very good results with a mean $R^2$ value of 0.988 for the validation sequences (Fig. 8) and we were able to predict the increase in RoM under cyclic loading sufficiently correct. This proves the network to be a good starting point for further studies improving the abilities to predict the mechanical behavior of spinal segments using ANN. When the model is trained and shows the desired results as mentioned above, it can predict the behavior of a spinal segment under cyclic loading it has not seen before. Therefore, neither time- and cost-expensive *in vitro* tests nor complex and computationally expensive *in silico* studies have to be performed.

The use of RNN has multiple advantages over fitting complex material models to the experimental data.^18,27^ Firstly, the computational cost is reduced immensely. Secondly, specimen-specific data can be used as input parameters, so that ”patient-specific” output data can be generated without calibrating new material parameters. Thirdly, the complexity of the model is unlimited and restricted only by the amount and quality of the training data, which allows the model to describe the change in RoM for the whole spinal segment including IVD, facet joints, and ligamentous structure.

Thereby, the parameterization process of the moment-RoM curves enabled the use of a network trained with only 116 training sequences. The use of the modified Boltzmann sigmoid function,^32^ which only needed 10 parameters to describe one hysteresis curve minimized the number of necessary input and output parameters of the network. Training the network with all data points as performed in other studies^20,21^ led in our case to a divergence of the training process. In order to predict the change in moment-RoM with changing loading rates, Stolworthy *et al*.^25^ also used a parameterization as preprocessing. They found a simple linear correlation between the fitting parameters and the loading rate, which was used to predict the change in hysteresis curve. Our study is based on a similar idea but includes various factors of influence like cycles of loading, testing temperature, testing time, and direction of loading. The additional complexity could be bypassed with an RNN to predict the changing RoM curve under cyclic loading. Rahmanpanah *et al*.^21^ were able to predict load-displacement curves on long bones under cyclic loading. This study used two in-series feedforward back-propagation ANNs with descriptive parameters of the specimen and the strains as inputs. This shows the feasibility of specimen-specific parameters as input parameters to further improve the networks’ accuracy. Especially for spinal segments with their high variety between segments and specimens in geometry and IVD material behavior, the inclusion of those parameters can further improve the predictions made. However, for this study no improvement in the results was seen, when adding specimen-specific input parameters, e.g. donor age, in preliminary studies. It was assumed, that this is caused by the limited specimen number and variety.

A limitation that the present model can face concerns the patterns that it learns during the training process. If the trained surrogate comes across new patterns, it becomes essential that we retrain the model with similar patterns. This will ensure an efficient performance over a diverse range of input.

The promising results of this RNN can be used as a starting point for further studies. In the present research, we restrict the prediction of the RNN for RoM curves for pure moment loading in the range of $(-7.5, 7.5)$ Nm. In subsequent steps, we can increase the domain of the surrogate response by collecting more experimental data with different load amplitudes, loading patterns, and different segmental levels and bolster its generalization capability. This development of an ANN describing the change in moment-RoM curves of spinal segments depending on their loading history is especially interesting for further understanding the complex biomechanical behavior of the human lumbar spine and its dependencies. In the future, further trained RNNs with higher general validity could help find risk factors in loading patterns of the spine for WMSD.

## Supplementary Information

Below is the link to the electronic supplementary material.

Supplementary file1 (CSV 1769 kb).
